# The relationship of smartphone addiction with psychological distress and neuroticism among university medical students

**DOI:** 10.1186/s40359-020-00466-6

**Published:** 2020-09-11

**Authors:** Leonard Yik-Chuan Lei, Muhd Al-Aarifin Ismail, Jamilah Al-Muhammady Mohammad, Muhamad Saiful Bahri Yusoff

**Affiliations:** Department of Medical Education, School of Medical Sciences, Universiti Sains Malaysia (USM), Health Campus, Kubang Kerian, Malaysia

**Keywords:** Smartphone addiction, Medical students, Psychological distress, Neuroticism

## Abstract

**Background:**

Smartphone plays a vital role in higher education as it serves as a device with multiple functions. Smartphone addiction was reported on the rise among college and university students. The addiction may result in unwanted consequences on their academic performance and psychological health. One factor that consistently relates to psychological distress and smartphone addiction is the neurotic personality trait. This study explored the relationship of smartphone addiction with psychological health and neuroticism among USM medical students.

**Methods:**

A cross-sectional study was carried out on medical students in a public medical school. DASS-21, the neuroticism-subscale of USMaP-i and SAS-SV were administered to measure psychological distress, neuroticism, and smartphone addiction of the medical students. Spearman correlation was performed to examine the correlation between smartphone addiction with psychological distress and neuroticism. Simple linear regression was performed to investigate relationship factors of smartphone addiction.

**Results:**

A total of 574 medical students participated in this study. The prevalence of smartphone addiction was 40.6%. It was higher among male (49.2%) compared to female (36.6%) medical students. The result showed a fair positive correlation between smartphone addiction and psychological health (rdepression = 0.277, *p*-value < 0.001; ranxiety = 0.312, p-value < 0.001; rstress = 0.329, p-value < 0.001). However, there was a poor positive correlation between smartphone addiction and neuroticism (r = 0.173, *p*-value < 0.001). The simple linear regression showed a significant increase in the levels of depression, anxiety, stress and neuroticism upon one unit increase in smartphone addiction (bdepression = 0.101, *p*-value < 0.001; banxiety = 0.120, p-value < 0.001; bstress = 0.132, p-value < 0.001; bneuroticism = 0.404, p-value < 0.05). These results indicated significant relationships between smartphone addiction, psychological health and neuroticism.

**Conclusion:**

This study suggested a high prevalence of smartphone addiction among medical students, particularly in male medical students. The smartphone addiction might lead to psychological problems and the most vulnerable group is the medical student with the neurotic personality trait.

## Background

Smartphones possess the means to enrich learning activities from medical education in undergraduate and postgraduate training [1]. For example, smartphones are used in the pursuit of finding solutions to patient care, improving lifelong medical education, and professional partners through the use of social media [2]. With the advent of smartphones, its uses in higher education cannot be ignored and need to be examined to explore the consequences of its application. Smartphones can be defined as a hand-held device built as a mobile computing platform with advanced computing ability and connectivity. It serves to combine the functions of portable media players, low-end compact digital cameras, pocket video cameras, and GPS navigation units [3]. Furthermore, smartphones are being used for more than just a phone but rather a device that provides multiple functions including surfing the internet, email, navigation, social networking, and games [4].

Smartphones are gaining increasing importance in health care and researchers and developers are enticed with their applications related to health [5]. These devices have multiple features that can be positively employed which include speedy access to information, enhanced organization, and instantaneous communication [6] and can with certainty be used to enhance education [7]. However, smartphone addiction is a vital issue in the global population with problems comprising physical difficulties like muscular pain and eye illnesses, and psychological difficulties such as auditory and tactile delusion [8].

The use of smartphones reached figures over 50% in the majority of developed countries [9]. Malaysian Communications and Multimedia Commission (MCMC) has reported that in Malaysia, 24.5 million users have access to the internet in 2016 [10]. Smartphone stays as the most popular gadget for users to enter the internet (89.4%) creating a mobile-oriented country [10]. Over the past several years, there has been an increasing amount of studies that explored smartphone addiction [11–14]. The bulk of these studies focused on smartphone addiction and its potential influences on individuals [15]. On top of that, among Malaysian medical schools, two studies showed the prevalence of at-risk cases of developing smartphone addiction: 46.9% in Universiti Putra Malaysia (UPM) [16] and 52.2% in Universiti Teknologi MARA (UiTM) [17]. Several studies have reported a high prevalence of smartphone addiction: the prevalence of smartphone addiction in a Malaysian medical school was 46.9% [16], Saudi Arabian university students was 48% [18], Saudi Arabian dental students was 71.9% [19] and Indian medical students was 85.4% [20]. Conversely, some studies have reported a low prevalence of smartphone addiction: the prevalence of smartphone addiction in Chinese medical college students was 29.8% [4], Saudi Arabian students was 33.2% [21] and Saudi Arabian medical students was 36.5% [22]. These results suggested more than a quarter of students in higher education experienced smartphone addiction that requires further exploration of possible factors contributing to it as well as its consequences on students’ wellbeing.

Previous studies have shown links between smartphone addiction and depression [23–25], smartphone addiction and anxiety [26–29], smartphone addiction and stress [11, 30], smartphone addiction and neuroticism [27, 31]. However, these studies are in the general population and university students. Our study is looking at the specific population of medical students in a public medical school. Also, to the best of our knowledge, there are no studies in medical students that links smartphone addiction, psychological distress, and neurotic traits. Medical students use smartphones to facilitate retrieving information resources [32–34]. There is almost universal ownership of smartphones among medical students [35]. The applications on smartphones in medical education have reported to increase student involvement, improve the feedback process, and enhance communication between student and teacher [36–39]. Instant messaging smartphone applications such as WhatsApp can be used as a method to facilitate communication and education among groups of medical students [40]. It is important to know the effect of the increased use of smartphones in relation to psychological distress among medical students.

The study of smartphone addiction among medical students become vital as it enlightens us on smartphone usage. In Malaysia, there have been numerous studies on smartphone addiction [41, 42]. However, there are not many studies done on smartphone addiction and its effects on medical undergraduates. Individuals measured with the personality trait that is high in neuroticism may be predisposed to addiction and behavioural problems [43]. Thus, we also decided to include the personality trait neuroticism as it is linked to addiction [44, 45], and we are interested in studying the relationship between neuroticism and smartphone addiction. Neuroticism is present among medical students [46]. Medical students with neurotic tendencies behave negatively to academic stress, and this becomes a contributing factor to low academic performance [46]. Students with neuroticism are more vulnerable to smartphone addiction which can lead to psychological distress. The current research may play a role in developing intervention measures such as behavioral therapies and counseling. It also may serve to help medical students improve their awareness of their emotionality and its effect on smartphone use. Therefore, the primary focus of this study is to investigate the relationship of smartphone addiction with psychological distress and neuroticism among medical students.

### Research hypothesis

1. The prevalence of smartphone addiction among USM medical students is more than 40%.
2. There is a significant correlation between smartphone addiction and depression among USM medical students.
3. There is a significant correlation between smartphone addiction and anxiety among USM medical students.
4. There is a significant correlation between smartphone addiction and stress among USM medical students.
5. There is a significant correlation between smartphone addiction and neuroticism among USM medical students.
6. Depression, anxiety, stress, and neuroticism significantly predict the smartphone addiction level.

## Methods

### Study sample

A cross-sectional study was carried out on year 1 to year 5 medical students in a public medical school in Malaysia. Before handing out the questionnaires, all students were informed about the study and their participation was voluntary. An informed consent to participate and publication was obtained from all participants. Bias was not explored in this study.

### Sampling method and calculation

The sample size was determined by the single proportion size formula. The initial sample size calculation was based on a pilot study that involved students and staff [17] and the largest sample size needed was 384. After taking into account, 30% drop-out rate, the total number of sample size was 384/ (1–0.3) = 548. All medical students were included, those who did not sign the consent form were excluded from the study.

### Data collection tools

There were three psychometric instruments used in this study; 1) Depression Anxiety Stress Scales (DASS-21); 2) modified USM Personality Inventory (USMaP-i); and 3) Smartphone Addiction Scale – Short Version (SAS-SV).

### DASS-21

The DASS instrument was first introduced by Lovibond and Lovibond (1995) and delivered a self-reporting measure, which was created to evaluate the features associated to anxiety, stress, and depression. For each DASS-21 subscale, the score must be multiplied by two to simulate the DASS-42 version: the range of score from 0 to 42. A high score in each subscale is equal to a high degree of symptoms [47]. In a validation study done, the internal consistency of each subscale was high ranging from 0.70 for the stress sub-scale to 0.88 for the overall scale. The scores on each of the three subscales and the combinations of two or three of them were able to identify mental disorders of depression and anxiety in women with a sensitivity of 79.1% and a specificity of 77% at the optimal cut off of > 33 [23].

### Modified USMaP-i

The USM Personality Inventory was created to measure the Big-Five Personality traits and is identified to be a reliable and valid instrument to evaluate the personality traits of prospective medical students [48]. This inventory was created explicitly to identify personal traits of Malaysian candidates who seek to apply to the medical course in USM [48]. The 15-item version of USMaP-i showed an acceptable level of internal consistency with each personality domain ranged from 0.64 and 0.84 as reported on the International Personality Item Pool Website [49]. We selected the 3 items that represent neuroticism to assess the neuroticism personality trait. Neuroticism is usually associated with features like depression, distress, anxiety, moodiness, poor coping ability, and sadness [50]. The total Cronbach alpha for the neuroticism subscale for USMaP-I is 0.722 [26, 49].

### SAS-SV

The smartphone addiction scale was first developed and validated by Kwon, Lee et al. (2013) as a way to evaluate smartphone addiction in teenagers. This scale has shown to be validated with high psychometric sound properties in various countries [3, 12, 16, 27, 51]. The Smartphone Addiction Scale - Short Version (SAS-SV) is a validated scale that consists of ten items in the questionnaire that are used to measure the levels of smartphone addiction [3]. The total score is from 10 to 60. The coefficient for Cronbach alpha correlation obtained is 0.91 for Smartphone Addiction Short Version [3]. The strength of SAS-SV is that it can be used to discern a potentially high-risk group for smartphone addiction, both in the educational field and community [3]. The cut-off point for significant smartphone addiction for male is 31 and female is 33 based on the recommendation by Kwon et al. (2013).

### Data collection

Data collection was performed via a self-guided questionnaire. Individuals were screened for one inclusion criteria and one exclusion criteria. Individuals who were medical students were eligible to participate (inclusion criteria). Individuals who were not willing to participate were not included in the study (exclusion criteria). Participants who submitted incomplete responses were excluded from this study.

### Ethical consideration

Ethical clearance was obtained from the Human Research Ethics Committee of USM (JEPeM) with study protocol code (USM/JEPeM/18070352). Signed consents were taken from medical students. Instructions and information about this study were given to them. Each medical student was given an ID for tracing and profiling purposes. They were informed that the results of this study will not affect their academic results in any way. The questionnaires were distributed to all medical students after lecture sessions.

### Statistical analysis

The data was analyzed using Statistical Package for Social Sciences (SPSS) version 24. Spearman correlation and simple linear regression tests were performed to examine the relationships of smartphone addiction with psychological distress and neuroticism. To accurately represent the relationship of smartphone addiction and neuroticism, the items in the modified USMaP-i were recoded due to negative items present in the neuroticism subscale: (Question 6 + Question 10 + Question 14). In the regression analysis, depression, anxiety, stress and neuroticism are independent variables and smartphone addiction is the dependent variable. A single regression analysis is used to account for the effects of multicollinearity because the correlation coefficient values of stress, depression, anxiety and neurotic tendencies are large. This research was not designed to investigate the gender differences and its correlations with depression, anxiety, stress and neuroticism. Further gender issues are not within the scope of this study.

## Results

### Response rate

The survey’s response rate was 83.9% (574 out of 674). There was a higher proportion of female medical students (68.5%) than male medical students (31.5%). Malay students were the majority (65.3%) followed by Chinese (16%), Indian (15.5%) and other races (3.1%). The majority of students were between 19 and 23 years old. The proportion of students in each year of study was more or less similar or equal in numbers. Medical students that did not participate in the survey for reasons of lack of interest, time constraints, and fatigue.

In this study, the prevalence of smartphone addiction found was 40.6%. There is a higher prevalence of male students addicted to smartphone (49.2%) compared to the female students (36.6%). The results of this analysis can be seen in Table 1.

**Table 1:** Prevalence of smartphone addiction among USM medical students

	Prevalence	
Smartphone addiction	N (%)	Total (%)
Male	89 (49.2)	233 (40.6)
Female	144 (36.6)	

Cut-off point for significant smartphone addiction for male is 31 and female is 33 based on recommendation [3]

### Correlation of smartphone addiction, psychological distress and neuroticism

The correlation analysis for smartphone addiction with psychological health and neuroticism is shown in Table 2. The result showed a fair positive correlation between smartphone addiction and psychological health among USM medical students (rdepression = 0.277, *p*-value < 0.001; ranxiety = 0.312, p-value < 0.001; rstress = 0.329, p-value < 0.001). However, there was a poor positive correlation between smartphone addiction and neuroticism (r = 0.173, *p*-value < 0.001).

**Table 2:** Correlation between smartphone addiction, psychological distress and neuroticism

Parameters	Smartphone addiction	
	r	p-value
Depression	0.277	< 0.001
Anxiety	0.312	< 0.001
Stress	0.329	< 0.001
Neuroticism	0.173	< 0.001

Spearman correlation was set at 95% confidence interval, p-value less than 0.05 was considered as a significant level

Correlation coefficient (r): poor = less than 0.25, fair = 0.26–0.50, good = 0.51–0.75 (good), and excellent = 0.76–1.00

Assumption was not met as normality of distribution was violated.

### Linear regression of smartphone addiction, psychological distress and neuroticism

The regression analysis for smartphone addiction with psychological health and neuroticism is shown in Table 3. The simple linear regression study showed a significant increase in depression, anxiety, stress and neuroticism levels upon one unit increase in smartphone addiction (bdepression = 0.101, *p*-value < 0.001; banxiety = 0.120, p-value < 0.001; bstress = 0.132, *p*-value < 0.001; bneuroticism = 0.404, p-value < 0.05). These results indicated significant relationships between smartphone addiction, psychological health and neuroticism. Smartphone addiction is a significant relationship factor of depression, anxiety and stress, while neuroticism is a significant relationship factor of smartphone addiction.

**Table 3:** The linear relationship between smartphone addiction and psychological distress

Parameters	Smartphone addiction	
	b	p-value
Depression	0.101	< 0.001
Anxiety	0.120	< 0.001
Stress	0.132	< 0.001
Neuroticism	0.404	< 0.050

Linear regression was set at 95% confidence interval, less than 0.05 was considered as a significant level

## Discussion

### Prevalence of smartphone addiction

The prevalence of smartphone addiction among USM medical students was 40.6%; hypothesis 1 assumes the prevalence of smartphone addiction among USM medical students is more than 40%. This study reported that there is a higher prevalence of smartphone addiction among male medical students compared to female medical students, which is similar to a few other studies [4, 52, 53]. Other studies found a higher prevalence of smartphone addiction in females compared to males [3, 12, 22, 54, 55]. Interestingly, previous studies [24, 25] did not report that gender is associated with smartphone addiction. The high prevalence of smartphone addiction (40.6%) in this study may be explained by smartphones becoming the main communication device among Malaysians and elsewhere. The percentage of smartphone consumers gradually rose from 68.7% in 2016 to 75.9% in 2017 [10]. Another observation is that medical students are using smartphones for social media messaging services such as WhatsApp and WeChat for communication purposes as well as for their studies, hence smartphones are becoming a vital tool in personal and professional life [56]. A study reported that WhatsApp assisted in easy learning and provided a way for clear communication of knowledge in shorter periods [57]. The higher prevalence of smartphone addiction in male medical students may be due to male medical students using their smartphone more for their entertainment such as online games while females use their smartphones for social interactions [58–60].

### Relationship between smartphone addiction and depression

This study found a significant and fair positive correlation (r = 0.277) between smartphone addiction and depression, as in research hypothesis 2. Likewise, previous studies among adults reported a strong positive correlation between smartphone addiction and depression [28]. Other findings further support the fact that high levels of smartphone addiction were correlated with depression [29]. In the Malaysian context, a study among university students showed students who had high scores of smartphone addiction reported high scores of depression [61] that suggests a relationship between smartphone addiction and depression. Another study found that the group with high smartphone use showed greater levels of depression compared to the low smartphone use group among university students in Turkey [12]. However, other studies have found no relationship between smartphone addiction and depression [62]. These facts consistently suggested a positive correlation between smartphone addiction and depression.

The regression analysis showed the increase of smartphone addiction scores leads to the increase of depression scores, indicating it is a relationship factor. These results are similar to previous findings, in which smartphone addiction was reported to be found as a predictor of depression for undergraduates in a local Malaysian university [61]. Another study also supports this finding, in which it reported the severity of smartphone use predicted depression [12]. Conversely, previous studies reported vice-versa whereby depression predicted smartphone addiction among university students [25, 30, 63]. These facts suggested that smartphone uses among university students should be considered as high-risk behaviour that negatively affects their psychological health. There are several possible explanations for our results. Individuals with mood disorders are more prone to become a smartphone addict [64]. Lemola et.al (2015) reported that using electronic media at night is associated with sleep disturbances and depressive symptoms. One study stated that individuals with lower levels of self-perceived health conditions and emotions tended to display an excessive use of smartphones [65]. This suggested that individuals were in a constant cycle of attempting to compensate for their perceived health status, without being fully aware that smartphone addiction has undesirable implications to their physical, emotional, and social well-being.

The relationship between smartphone addiction and depression is evident in this study and shows that medical students that have smartphone addiction are at risk of having depression. Medical students displaying high levels of smartphone addiction and depression should be observed and given help if necessary. This can be done by promoting the responsible usage of smartphone use among medical students in activities. Sensible usage of smartphones is suggested, especially on younger adults who could be at greater risk of depression [28].

### Relationship between smartphone addiction and anxiety

The results of our study indicate that there was a fair positive correlation between (r = 0.312) smartphone addiction and anxiety, as in research hypothesis 3. Demirci et, al. (2015) has found that smartphone use severity was positively correlated with anxiety and that corresponds with the findings in our study. Several other studies describe smartphone addictions are reported to increase with anxiety levels [54, 55, 66, 67].

Our regression analysis revealed that increased smartphone addiction scores are a significant relationship factor in increased anxiety scores. Demirci et, al. (2015) reported that smartphone use severity predicted anxiety and it is consistent with our findings. A study reported that smartphone addiction was reported to be found as a predictor of anxiety in Malaysian undergraduate students [61]. In contrast, previous studies reported that anxiety significantly predicted smartphone addiction [25, 31, 63].

A possible explanation for our results is medical students may habitually check their smartphones in the likelihood of reducing their anxiety by receiving assurance through messages from their friends. The pattern of an individual checking his or her phone and receiving notifications also function in getting social reassurance behaviour from friends [68]. This behaviour of seeking reassurance can generally include symptoms of loneliness, depression, and anxiety that is the driving factor for reassurance seeking [68].

### Relationship between smartphone addiction and stress

The results of this study indicate that there was a fair positive correlation between (r = 0.329) smartphone addiction and stress, as in research hypothesis 4. In another important finding, Samaha et, al. (2016) show the results between the risk of smartphone and perceived stress, reporting a slight positive correlation with an elevated risk of smartphone addiction associated with elevated levels of perceived stress which supports our study. Previous studies reported that stress leads to smartphone use [56, 69], while another study proposes that smartphone use may cause stress [70]. In our regression analysis, increased smartphone addiction scores are a significant relationship factor in increased stress scores. Conversely, in a sample of Taiwanese university students reported a positive predictive effect of family and emotional stresses on smartphone addiction [11].

There are several explanations for the study results. Medical students are under stressful medical training [71], therefore they are prone to being under stress which in turn lowers self-control which may increase their chances of smartphone addiction. Smartphone addiction is influenced by self-control [72]. Self-control is defined as the capacity to alter one’s responses, such as overriding some impulses to bring behavior in line with goals and standards [73]. According to Cho et, al. (2017), an increase in stress degree results in a lowered self-control ability, and reduction in self-control further increases the chances of smartphone addiction.

### Relationship between smartphone addiction and neuroticism

In this study, the results indicate that there is a poor positive correlation between (r = 0.173) smartphone addiction and neuroticism, as in research hypothesis 5. Neuroticism was reported to be significantly related to excessive use of smartphones [55] and corresponded with the findings of our study. These results are similar to other study findings reported that individuals who possessed high levels of neuroticism also report a high level of smartphone addiction [74]. In another study, neuroticism predicts problematic smartphone use [75]. However, the study [76] did not report a significant relationship between neuroticism and problematic phone use. In our regression analysis, increased neuroticism scores are a significant relationship factor in increased smartphone addiction scores. It was found neurotic personality increased the degree of smartphone addiction [55]. Problematic mobile use is positively associated with neuroticism [77]. There are several explanations for this result, medical students may be more vulnerable to smartphone addiction. The neuroticism trait has been linked to smartphone addiction [78, 79]. A study stated that neuroticism is associated with a chain-mediating effect with smartphone addiction and depression, all vital variables that deteriorate the quality of life [80]. Apart from that, another study showed that there is a positive relationship between neuroticism and smartphone use while driving [81]. Another possible explanation is that medical students with neuroticism may depend on their smartphones to get reassurance from their friends. Individuals with high neuroticism tend to use their smartphones to get emotional and social reassurance from their relationships [82].

### Implications and future research

Depression, anxiety, stress and neuroticism significantly predict smartphone addiction level, as in research hypothesis 6. The results show that there is a high prevalence of smartphone addiction among medical students. This means that a large proportion of students are affected by smartphone over usage and suggests a widespread occurrence that needs to be addressed by all relevant parties. It raises a deep concern because academic performances may be affected by a large number of medical students with smartphone addiction. As a consequence of smartphone addiction, individuals with smartphone addiction might meet with difficulties such as interpersonal adjustments, managing time, and academic performance [83]. This might affect the performance of the medical school as a whole in terms of academic results. The high prevalence of smartphone addiction in this study shows that they are at risk to have problems. Medical students displaying high levels of smartphone addiction should be monitored and given further help. Prevention is better than cure, thus smartphone addiction among medical students is recommended for early detection so that appropriate interventions can be planned accordingly. We also have to take into account the high prevalence of male medical students. Several approaches can be suggested to medical students who require further help for smartphone addiction; namely cognitive-behavioral approach, motivational interviewing, and behavioural cognitive treatment [84]. The implications for the intervention from the results of the study are to provide a baseline for research incorporating approaches tailored for medical students with smartphone addiction. This should address the most vulnerable group of students with the neuroticism personality trait.

### Limitations and future research

Considering limited undergraduate smartphone addiction studies in the local setting, the results reported in this study provide insights into the professional health care team. It should be noted that smartphone usage is culturally bound experience and will contrast across countries with varying degrees of technology availability and advances in that region. This study does not report cause and effect relationships. Confounding variables were not studied. For example, in the curriculum at the medical school, each student has to go through e-learning (teaching and learning activities) and assessment that requires smartphone use. This suggests that in reality the medical students may be tasked with activities that require them to use their smartphones for education purposes, i.e. hours spent on smartphones for assignments and lectures. Further research can build upon our findings and investigate screening and interventions for smartphone addiction among medical students.

## Conclusions

This study found the prevalence of smartphone addiction among medical students was high, particularly in male medical students. The smartphone addiction might lead to psychological problems and the most vulnerable group was students with the neuroticism personality trait. Thus, there is a need to create and implement programs to promote healthy smartphone usage to minimize the impact of smartphone addiction on psychological health. By doing so, one may implement effective intervention and prevention strategies to groups of students with smartphone addiction. We believe that with a proper guidance; students may be able to use their smartphones more responsibly.

## Data Availability

Data generated and analysed during the current study are not publicly available as individual privacy could be comprised; however, may be available from the corresponding author on reasonable request and with the permission of the medical school.

## Abbreviations

DASS-21: Depression Anxiety Stress Scale
JEPeM: Human Research Ethics Committee of University Sains Malaysia
MCMC: Malaysian Communication and Multimedia Commission
SAS-SV: Smartphone Addiction Scale Short Version
SMS: School of Medical Sciences
UiTM: Universiti Teknologi MARA
UPM: Universiti Putra Malaysia
USM: Universiti Sains Malaysia
USMaP: iUniversiti Sains Malaysia Personality Inventory

## References

[1] Masters K, Ellaway RH, Topps D, Archibald D, Hogue RJ (2016). Mobile technologies in medical education: AMEE guide no. 105. Med Teach.

[2] Joshi N, Lin M (2013). The smartphone: how it is transforming medical education, patient care, and professional collaboration. African J Emergency Med.

[3] Kwon M, Kim D-J, Cho H, Yang S (2013). The smartphone addiction scale: development and validation of a short version for adolescents. PLoS One.

[4] Chen B, Liu F, Ding S, Ying X, Wang L, Wen Y (2017). Gender differences in factors associated with smartphone addiction: a cross-sectional study among medical college students. BMC Psychiatry.

[5] Sandholzer M, Deutsch T, Frese T, Winter A (2015). Predictors of students’ self-reported adoption of a smartphone application for medical education in general practice. BMC Med Educ.

[6] Robinson T, Cronin T, Ibrahim H, Jinks M, Molitor T, Newman J (2013). Smartphone use and acceptability among clinical medical students: a questionnaire-based study. J Med Syst.

[7] Almunawar MN, Anshari M, Susanto H, Chen CK (2015). Revealing customer behavior on smartphones. Int J Asian Business Information Manage (IJABIM).

[8] De-Sola J, Talledo H, Rubio G, de Fonseca FR (2017). Development of a mobile phone addiction craving scale and its validation in a Spanish adult population. Front Psych.

[9] Van Deursen AJ, Bolle CL, Hegner SM, Kommers PA (2015). Modeling habitual and addictive smartphone behavior: the role of smartphone usage types, emotional intelligence, social stress, self-regulation, age, and gender. Comput Hum Behav.

[10] Malaysian Communications And Multimedia Comission (2017). Hand phone users Survery 2017.

[11] Chiu S-I (2014). The relationship between life stress and smartphone addiction on Taiwanese university student: a mediation model of learning self-efficacy and social self-efficacy. Comput Hum Behav.

[12] Demirci K, Akgönül M, Akpinar A (2015). Relationship of smartphone use severity with sleep quality, depression, and anxiety in university students. J Behav Addict.

[13] Ghosh A, Jha R, Malakar SK (2016). Pattern of smartphone use among MBBS students in an Indian medical college. IJAR..

[14] Gowthami S, Kumar S (2016). Impact of smartphone: a pilot study on positive and negative effects. Int J Scientific Eng Appl Sci (IJSEAS).

[15] Toda M, Monden K, Kubo K, Morimoto K (2006). Mobile phone dependence and health-related lifestyle of university students. Soc Behav Personal Int J.

[16] Ching SM, Yee A, Ramachandran V, Lim SMS, Sulaiman WAW, Foo YL (2015). Validation of a Malay version of the smartphone addiction scale among medical students in Malaysia. PLoS One.

[17] Nikmat AW, Hashim NA, Saidi MF, Zaki NSM, Shukri NNH, Abdulla NB (2018). The use and addiction to smart phones among medical students and staffs in a public University in Malaysia. Asean J Psychiatry.

[18] Aljomaa SS, Qudah MFA, Albursan IS, Bakhiet SF, Abduljabbar AS (2016). Smartphone addiction among university students in the light of some variables. Comput Hum Behav.

[19] Venkatesh E, Al Jemal MY, Al Samani AS. Smart phone usage and addiction among dental students in Saudi Arabia: a cross sectional study. Int J Adolesc Med Health. 2017;31(1). 10.1515/ijamh-2016-0133. 10.1515/ijamh-2016-0133 28384117

[20] Sethuraman AR, Rao S, Charlette L, Thatkar PV, Vincent V (2018). Smartphone addiction among medical college students in the Andaman and Nicobar Islands. Int J Commun Med Public Health.

[21] Qudah MFA, Albursan IS, Bakhiet SFA, Hassan EMAH, Alfnan AA, Aljomaa SS, et al. Smartphone Addiction and Its Relationship with Cyberbullying Among University Students. Int J Ment Health Ad. 2019;17(3):628–43.

[22] Alhazmi AA, Alzahrani SH, Baig M, Salawati EM, Alkatheri A (2018). Prevalence and factors associated with smartphone addiction among medical students at king Abdulaziz University, Jeddah.

[23] Tran TD, Tran T, Fisher J (2013). Validation of the depression anxiety stress scales (DASS) 21 as a screening instrument for depression and anxiety in a rural community-based cohort of northern Vietnamese women. BMC Psychiatry.

[24] Alosaimi FD, Alyahya H, Alshahwan H, Mahyijari NA, Shaik SA (2016). Smartphone addiction among university students in Riyadh, Saudi Arabia. Saudi Med J.

[25] Matar Boumosleh J, Jaalouk D (2017). Depression, anxiety, and smartphone addiction in university students- a cross sectional study. PLoS One.

[26] Yusoff MSB, Rahim AFA, Aziz RA, Pa MNM, Mey SC, Ja'afar R (2011). The validity and reliability of the USM personality inventory (USMaP-i): its use to identify personality of future medical students. Intern Med J.

[27] Kwon M, Lee J-Y, Won W-Y, Park J-W, Min J-A, Hahn C (2013). Development and validation of a smartphone addiction scale (SAS). PLoS One.

[28] Alhassan AA, Alqadhib EM, Taha NW, Alahmari RA, Salam M, Almutairi AF (2018). The relationship between addiction to smartphone usage and depression among adults: a cross sectional study. BMC Psychiatry.

[29] Bian M, Leung L (2015). Linking loneliness, shyness, smartphone addiction symptoms, and patterns of smartphone use to social capital. Soc Sci Comput Rev.

[30] M-o K, Kim H, Kim K, Ju S, Choi J, Yu M (2015). Smartphone addiction: (focused depression, aggression and impulsion) among college students. Indian J Sci Technol.

[31] Hong ENC, Hao LZ, Kumar R, Ramendran C, Kadiresan V (2012). An effectiveness of human resource management practices on employee retention in institute of higher learning: a regression analysis. Int J Bus Res Manag.

[32] Jamal A, Temsah M-H, Khan SA, Al-Eyadhy A, Koppel C, Chiang MF (2016). Mobile phone use among medical residents: a cross-sectional multicenter survey in Saudi Arabia. JMIR Mhealth Uhealth.

[33] Robinson R (2015). Spectrum of tablet computer use by medical students and residents at an academic medical center. PeerJ..

[34] Pulijala Y, Ma M, Ju X, Benington P, Ayoub A (2016). Efficacy of three-dimensional visualization in mobile apps for patient education regarding orthognathic surgery. Int J Oral Maxillofac Surg.

[35] Browne G, O’Reilly D, Waters C, Tummon O, Devitt D, Stewart B (2015). Smart-phone and medical app use amongst Irish medical students: a survey of use and attitudes. BMC proceedings.

[36] Cochrane T, editor Mobile social media as a catalyst for pedagogical change. EdMedia+ innovate learning; 2014: Association for the Advancement of computing in education (AACE).

[37] Makoe M (2010). Exploring the use of MXit: a cell-phone social network to facilitate learning in distance education. Open Learning.

[38] Nicholson S (2002). Socialization in the “virtual hallway”: instant messaging in the asynchronous web-based distance education classroom. Internet High Educ.

[39] Rambe P, Bere A (2013). Using mobile instant messaging to leverage learner participation and transform pedagogy at a S outh a frican U niversity of T echnology. Br J Educ Technol.

[40] Raiman L, Antbring R, Mahmood A (2017). WhatsApp messenger as a tool to supplement medical education for medical students on clinical attachment. BMC Med Educ.

[41] Parasuraman S, Sam AT, Yee SWK, Chuon BLC, Ren LY (2017). Smartphone usage and increased risk of mobile phone addiction: a concurrent study. Int J Pharmaceutical Investigation.

[42] Hakkı B, Muhammed FP (2018). Investigating the smart phone addictions of vocational school students from different variables. Malaysian Online J Educ Technol.

[43] Shaffer HJ, Hall MN, Vander BJ (2000). "computer addiction": a critical consideration. Am J Orthopsychiatry.

[44] Andreassen CS, Griffiths MD, Gjertsen SR, Krossbakken E, Kvam S, Pallesen S (2013). The relationships between behavioral addictions and the five-factor model of personality. J Behav Addict.

[45] Tsai HF, Cheng SH, Yeh TL, Shih C-C, Chen KC, Yang YC (2009). The risk factors of internet addiction—a survey of university freshmen. Psychiatry Res.

[46] Bhagat V, Nayak RD (2014). Neuroticism and academic performance of medical students. Int J Humanit Soc Sci Invent.

[47] Lovibond SH, Lovibond PF (1995). Psychology Foundation of a. manual for the depression anxiety stress scales.

[48] Yusoff MSB (2013). Stability of the USMaP-i in measuring the big five personality traits. Intern Med J.

[49] Yusoff MSB (2013). Construct validity, internal consistency and normative data of the USMaP-i in a sample of medical students.

[50] Goldberg LR, Johnson JA, Eber HW, Hogan R, Ashton MC, Cloninger CR (2006). The international personality item pool and the future of public-domain personality measures. J Res Pers.

[51] De Pasquale C, Sciacca F, Hichy Z (2017). Italian validation of smartphone addiction scale short version for adolescents and young adults (SAS-SV). Psychology..

[52] Chóliz M (2012). Mobile-phone addiction in adolescence: the test of mobile phone dependence (TMD). Progress Health Sci.

[53] Dixit S, Shukla H, Bhagwat A, Bindal A, Goyal A, Zaidi AK (2010). A study to evaluate mobile phone dependence among students of a medical college and associated hospital of Central India. Indian J Commun Med.

[54] Choi S-W, Kim D-J, Choi J-S, Ahn H, Choi E-J, Song W-Y (2015). Comparison of risk and protective factors associated with smartphone addiction and internet addiction. J Behav Addict.

[55] Mok J-Y, Choi S-W, Kim D-J, Choi J-S, Lee J, Ahn H (2014). Latent class analysis on internet and smartphone addiction in college students. Neuropsychiatr Dis Treat.

[56] Wang J-L, Wang H-Z, Gaskin J, Wang L-H (2015). The role of stress and motivation in problematic smartphone use among college students. Comput Hum Behav.

[57] Barhoumi C (2015). The effectiveness of WhatsApp Mobile learning activities guided by activity theory on Students' knowledge management. Contemp Educ Technol.

[58] Cooper A, Morahan-Martin J, Mathy RM, Maheu M (2002). Toward an increased understanding of user demographics in online sexual activities. J Sex Marital Therapy.

[59] Fattore L, Melis M, Fadda P, Fratta W (2014). Sex differences in addictive disorders. Front Neuroendocrinol.

[60] Johansson A, Götestam KG (2004). Internet addiction: characteristics of a questionnaire and prevalence in Norwegian youth (12–18 years). Scand J Psychol.

[61] Ithnain N, Ezzat Ghazali S, Jaafar N (2018). Relationship between smartphone addiction with anxiety and depression among undergraduate students in Malaysia.

[62] Lemola S, Perkinson-Gloor N, Brand S, Dewald-Kaufmann JF, Grob A (2015). Adolescents’ electronic media use at night, sleep disturbance, and depressive symptoms in the smartphone age. J Youth Adolesc.

[63] Aker S, Sahin MK, Sezgin S, Oguz G (2017). Psychosocial factors affecting smartphone addiction in university students. J Addict Nurs.

[64] Zhang KZ, Chen C, Lee MK (2014). Understanding the role of motives in smartphone addiction.

[65] Kim HJ, Min JY, Min KB. Association between psychological and self-assessed health status and smartphone overuse among Korean college students. J Ment Health. 2019;28(1):11–6. 10.1080/09638237.2017.1370641 28868959

[66] Hwang K-H, Y-s Y, Cho O-H (2012). Smartphone overuse and upper extremity pain, anxiety, depression, and interpersonal relationships among college students. J Korea Contents Assoc.

[67] Haug S, Castro RP, Kwon M, Filler A, Kowatsch T, Schaub MP (2015). Smartphone use and smartphone addiction among young people in Switzerland. J Behav Addict.

[68] Billieux J, Maurage P, Lopez-Fernandez O, Kuss DJ, Griffiths MD (2015). Can disordered mobile phone use be considered a behavioral addiction? An update on current evidence and a comprehensive model for future research. Curr Addict Rep.

[69] Jeong S-H, Kim H, Yum J-Y, Hwang Y (2016). What type of content are smartphone users addicted to?: SNS vs. games. Comput Hum Behav.

[70] Murdock KK (2013). Texting while stressed: implications for students’ burnout, sleep, and well-being. Psychol Pop Media Cult.

[71] Yusoff MSB, Rahim AFA, Yaacob MJ (2010). Prevalence and sources of stress among Universiti Sains Malaysia medical students. Malaysian J Medical Sci.

[72] Kim N, Lee K (2012). Effects of self-control and life stress on smart phone addiction of university students. J Korea Soc Health Informatics Stat.

[73] Baumeister RF, Heatherton TF, Tice DM (1994). Losing control: how and why people fail at self-regulation: academic press.

[74] Toda M, Ezoe S, Mure K, Takeshita T (2016). Relationship of smartphone dependence to general health status and personality traits among university students. Open J Prev Med.

[75] Horwood S, Anglim J (2018). Personality and problematic smartphone use: a facet-level analysis using the five factor model and HEXACO frameworks. Comput Hum Behav.

[76] Bianchi A, Phillips JG (2005). Psychological predictors of problem mobile phone use. Cyberpsychol Behav.

[77] Augner C, Hacker GW (2012). Associations between problematic mobile phone use and psychological parameters in young adults. Int J Public Health.

[78] Pearson C, Hussain Z. Smartphone Use, Addiction, Narcissism, and Personality: A Mixed Methods Investigation. Int J Cyber Behav Psychol Learn. 2015;5:17–32.

[79] Roberts JA, Pullig C, Manolis C (2015). I need my smartphone: a hierarchical model of personality and cell-phone addiction. Personal Individ Differ.

[80] Gao T, Xiang Y-T, Zhang H, Zhang Z, Mei S (2017). Neuroticism and quality of life: multiple mediating effects of smartphone addiction and depression. Psychiatry Res.

[81] Luria G (2018). The mediating role of smartphone addiction on the relationship between personality and young drivers' smartphone use while driving. Transport Res F: Traffic Psychol Behav.

[82] Kim J-H, Seo M, David P (2015). Alleviating depression only to become problematic mobile phone users: can face-to-face communication be the antidote?. Comput Hum Behav.

[83] Hong F-Y, Chiu S-I, Huang D-H (2012). A model of the relationship between psychological characteristics, mobile phone addiction and use of mobile phones by Taiwanese university female students. Comput Hum Behav.

[84] Kim H (2013). Exercise rehabilitation for smartphone addiction. J Exercise Rehabilitation.

